# Automated quantitative multiplex immunofluorescence *in situ* imaging identifies phospho-S6 and phospho-PRAS40 as predictive protein biomarkers for prostate cancer lethality

**DOI:** 10.1186/1477-5956-12-40

**Published:** 2014-07-12

**Authors:** Michail Shipitsin, Clayton Small, Eldar Giladi, Summar Siddiqui, Sibgat Choudhury, Sadiq Hussain, Yi E Huang, Hua Chang, David L Rimm, David M Berman, Thomas P Nifong, Peter Blume-Jensen

**Affiliations:** 1 Metamark Genetics Inc, Cambridge, MA, USA; 2 Department of Pathology, Yale University Medical School, New Haven, CT, USA; 3 Department of Pathology and Molecular Medicine, Queen’s University, Kingston, ON, Canada; 4 Current address: Atreca, San Carlos, CA, USA; 5 Current address: Moderna, Cambridge, MA, USA; 6 Current address: XTuit Pharmaceuticals, Inc, Cambridge, MA, USA

**Keywords:** Prostate cancer, Biomarkers, Protein activity states, Quantitative multiplex immunofluorescence, Object recognition

## Abstract

**Background:**

We have witnessed significant progress in gene-based approaches to cancer prognostication, promising early intervention for high-risk patients and avoidance of overtreatment for low-risk patients. However, there has been less advancement in protein-based approaches, even though perturbed protein levels and post-translational modifications are more directly linked with phenotype. Most current, gene expression-based platforms require tissue lysis resulting in loss of structural and molecular information, and hence are blind to tumor heterogeneity and morphological features.

**Results:**

Here we report an automated, integrated multiplex immunofluorescence *in situ* imaging approach that quantitatively measures protein biomarker levels and activity states in defined intact tissue regions where the biomarkers of interest exert their phenotype. Using this approach, we confirm that four previously reported prognostic markers, PTEN, SMAD4, CCND1 and SPP1, can predict lethal outcome of human prostate cancer. Furthermore, we show that two PI3K pathway-regulated protein activities, pS6 (RPS6-phosphoserines 235/236) and pPRAS40 (AKT1S1-phosphothreonine 246), correlate with prostate cancer lethal outcome as well (individual marker hazard ratios of 2.04 and 2.03, respectively). Finally, we incorporate these 2 markers into a novel 5-marker protein signature, SMAD4, CCND1, SPP1, pS6, and pPRAS40, which is highly predictive for prostate cancer-specific death. The ability to substitute PTEN with phospho-markers demonstrates the potential of quantitative protein activity state measurements on intact tissue.

**Conclusions:**

In summary, our approach can reproducibly and simultaneously quantify and assess multiple protein levels and functional activities on intact tissue specimens. We believe it is broadly applicable to not only cancer but other diseases, and propose that it should be well suited for prognostication at early stages of pathogenesis where key signaling protein levels and activities are perturbed.

## Background

While tests for recurrent, validated gene mutations have great prognostic and predictive value [1-5], these mutations are relatively rare in early stage cancers. Multivariate gene-based tests require homogenized tissue with variable ratios of tumor and benign tissue resulting in less accurate biomarker measurements [6,7]. In these types of tests, phenotype must be inferred from genetic and mutational patterns. In contrast, direct *in situ* measurement of protein levels and post-translational modifications should more directly reflect the status of oncogenic signaling pathways. Thus, it is reasonable to expect a protein-based approach to be highly valuable for prognostication.

A number of other issues complicate prognostic testing. In prostate cancer, tumor heterogeneity is pronounced, and sampling error can contribute to incorrect predictions. Pathologist discordance in Gleason grading and tumor staging also renders prognostication in this multifocal disease difficult. In an attempt to address these shortcomings, we set out to develop an automated quantitative multiplex immunofluorescence imaging approach for intact tissue that integrates morphological object recognition and molecular biomarker measurements from defined, relevant tissue regions at the individual slide level where the quantitative nature of the signal intensity is positively correlated with the amount of protein accessible on the tissue. We used this system to predict lethal outcome from radical prostatectomy tissue using four previously reported markers, PTEN, SMAD4, CCND1 and SPP1 [8]. Importantly, we also demonstrate that quantitative measurements of protein activity states reflective of PI3K/AKT and mitogen-activated protein kinase (MAPK) signaling status, specifically pPRAS40 and pS6, are predictive of prostate cancer lethal outcome based on univariate and multivariate analyses. As such, they can substitute for PTEN, a highly validated prognostic marker which itself regulates PI3K/AKT pathway signaling [9-13]. Together these data identify a 5 marker novel lethal outcome predictive signature consisting of SMAD4, CCND1, SPP1, pPRAS40 and pS6.

## Results

### Platform development

In order to develop an automated multiplex immunofluorescence imaging platform several technical requirements had to be met: 1) ability to quantitate multiple markers in a defined region of interest (i.e. in tumor versus surrounding benign tissue), 2) rigorous tissue quality controls, 3) balanced multiplex assay staining format, and 4) experimental reproducibility.

**Figure 1:**
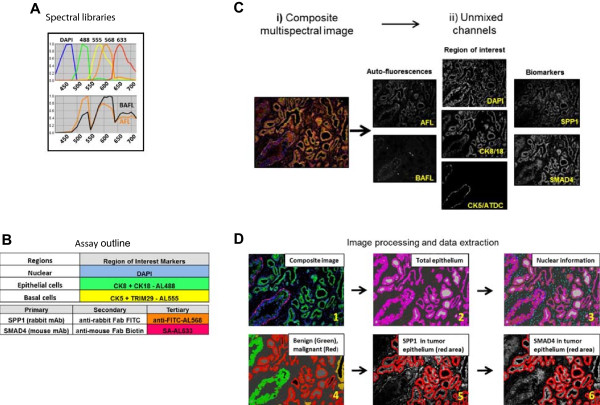
**Outline of experimental approach for automated, quantitative multiplex immunofluorescence and biomarker measurements in defined regions of interest of prostatectomy tissue. A)** Spectral profiles of each fluorophore in the spectral library used in the assay and profiles for tissue autofluorescence signals (AFL) and bright autofluorescence (BAFL) signals, respectively. **B)** A general outline of staining procedure for quantitative multiplex immunofluorescent biomarker measurements in tissue region of interest. SPP1 and SMAD4 were used as an example. Region of interest marker antibodies (KRT8 (CK8) and KRT18 (CK18) for total epithelium and KRT5 (CK5) and TRIM29 for basal epithelium) were directly conjugated to Alexa488 and Alexa 555, respectively. Biomarker antibodies were detected with a sequence of secondary and tertiary antibodies, as described. Colors in the table illustrate unique spectral positions of emission peaks for the indicated Alexa fluorophore dyes. **C)** A composite multispectral image (i) is unmixed into separate channels corresponding to AFL and BAFL, region of interest markers, and biomarkers, as indicated (ii). **D)** Definiens script-based tissue segmentation and biomarker quantitation. Moving through panels 1-6, from the composite image (1), first total epithelial regions are identified (2), followed by nuclear areas (3). The epithelial regions are further segmented into tumor shown in red, benign in green, and undetermined in yellow (4). Gray color denotes non-epithelial regions, *e.g.* stroma and vessels (4). Finally, biomarkers are quantified from tumor epithelium areas only, outlined in red (5 and 6).

To address the first, we optimized long-pass diamidino-2-phenylindole (DAPI), fluorescein isothiocyanate (FITC), tetramethylrhodamine isothiocyanate (TRITC) and indodicarbocyanine (Cy5) filter sets to have sufficient excitation energy and emission bandpass with minimal interference between channels. We further separated biomarker signals from endogenous autofluorescence through spectral unmixing of images (Figure 1A [14]). In order to measure biomarkers in tumor epithelium only, we needed to achieve “tissue segmentation”, distinguishing tumor from benign areas. Segmentation was achieved using a combination of feature extraction and protein co-localization algorithms. Total epithelium was stained using Alexa488 conjugated anti-KRT8 and KRT18 antibodies, while Alexa555 conjugated anti-KRT5 and TRIM29 antibodies stained basal epithelium (Figure 1B) [15,16]. Using automated Definiens (Munich, Germany) image analysis, epithelial structures with an outer layer of basal cells were considered benign, while those lacking basal cells were considered cancer [16]. Non-epithelial areas were considered stroma. Ultimately, quantitative biomarker values that correlated with accessible protein were extracted only from cancer epithelium (the ‘region of interest’; Figure 1B-D).

**Figure 2:**
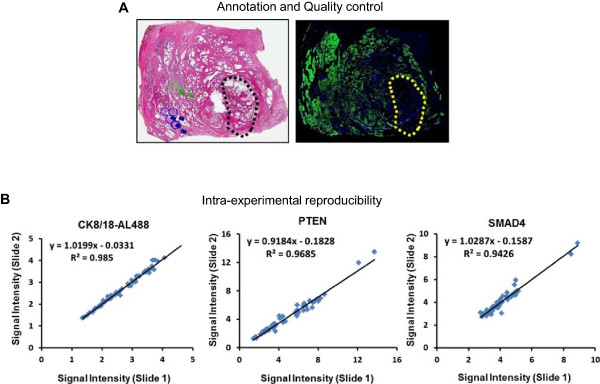
**Tissue annotation, quality control procedures and assay reproducibility. A)** Tissue annotation and quality control procedures. Left: A representative H&E-stained section of a human prostatectomy sample showing four (blue) and two (green) 1 mm diameter circles placed over the regions with the highest and lowest Gleason pattern, respectively, as annotated by an expert pathologist. Two cores (1 mm diameter each) were taken from two of the four blue regions to generate TMA blocks. Right: A consecutive section of the same prostatectomy sample was stained with DAPI and KRT8/KRT18-Alexa488. Areas with bright staining of prostate epithelium by KRT8/18 cytokeratin antibodies were considered good quality regions, while areas with little or no staining (as indicated within the yellow punctate area) were considered of low quality and not deemed suitable for TMA construction. **B)** Intra-experimental reproducibility. Two consecutive sections from a prostate tumor test TMA were stained in the same experiment. Images were acquired using the Vectra system and processed with a Definiens script. Scatter plots compare mean values of KRT8/18, PTEN, and SMAD4 staining intensities from the same cores of the consecutive TMA sections. Linear regression curves, equations, and R^2^ values were generated using Excel software.

To evaluate tissue sample quality for study inclusion, we assessed staining intensities of several protein markers in benign tissue. Examination of a large number of prostate tissue blocks of variable quality revealed that KRT8, KRT18 and pSTAT3 (STAT3-phosphothreonine 705) intensities in benign epithelial regions and capillary endothelium, respectively, varied from ‘high’ to ‘low’ or ‘absent’ , according to tissue quality. On this basis, we categorized formalin-fixed, paraffin-embedded (FFPE) prostate cancer tissue blocks into four quality groups (Figure 2A and Additional file 1: Table S1). Only blocks from the best two groups were used to generate tumor microarray blocks (TMA), thereby controlling for specimen degradation and variability due to pre-analytic variation [17-19]. In total, we procured and tested 508 unique prostatectomy samples with lethal outcome annotation available (Folio Biosciences, Powell, OH). Of these, 418 passed quality testing and were used for our TMA (Additional file 1: Table S2).

To balance biomarker signal levels in our multiplex assay format, proteins with high expression levels, like cytokeratins and TRIM29 were visualized with directly conjugated antibodies, while biomarkers with lower expression levels required signal amplification through use of secondary and tertiary antibodies. Using a test prostate TMA containing low- and high-grade tumor material, dilutions of each antibody were optimized to minimize background and maximize specificity, and to ensure a dynamic range of at least 3-fold difference between low and high signal values (Figure 2B). Signals from consecutive TMA sections showed high reproducibility with typical R^2^ correlation values above 0.9 and differences in absolute values typically less than 10% (Figure 2B and data not shown).

### Ability to predict lethal outcome

**Figure 3:**
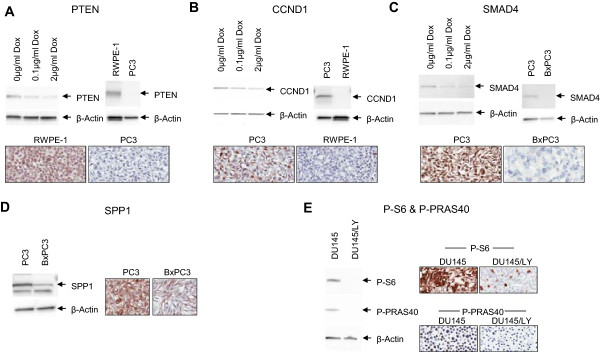
**Validation of PTEN, CCND1, SMAD4, SPP1, pS6 and pPRAS40 antibody specificity.** Doxycycline-inducible shRNA knockdown cell lines were established for PTEN **(A)**, CCND1 **(B)** and SMAD4 **(C)**. Doxycycline treatment reduced the abundance of the target protein in all cases as assessed by WB. Cell lines with high or low/negative levels of expression of PTEN **(A)**, CCND1 **(B)** and SMAD4 **(C)** were also examined by WB and IHC to further validate the specificity of the antibodies. SPP1 **(D)** antibody detected an SPP1-specific band and an additional band at a lower molecular weight as assessed by WB in PC3 cells, while the SPP1-specific upper band was significantly decreased in low SPP1-expressing BxPC3 cells. The staining intensity of the SPP1 antibody in PC3 and BxPC3 cells by IHC correlated well with the relative intensity of the SPP1-specific band detected by WB. The specificity of pS6 and pPRAS40 antibodies **(E)** was validated in DU145 cells. LY294002 treatment significantly reduced phosphorylation of RPS6 and AKT1S1 (PRAS40), as shown by WB and IHC, respectively.

**Figure 4:**
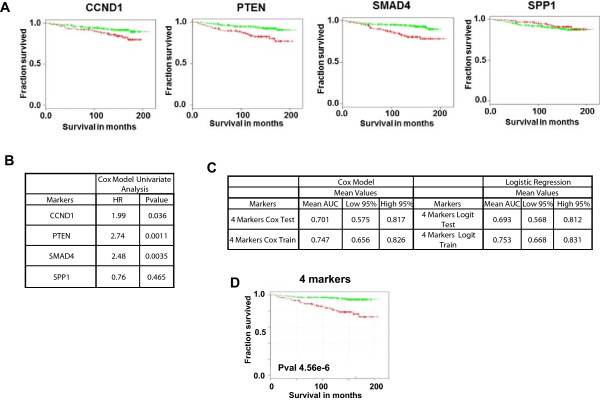
**QMIF assay performance with known 4-markers prognostic signature. A)** Kaplan-Meier curves for survival as a function of single biomarker protein expression in the study cohort. **B)** Univariate survival analysis. The population with the top 1/3 of risk score values was separated from the population with the bottom 2/3 of risk scores. P values (P) and HR are annotated. **C)** Multivariate Cox regression and logistic regression analyses of survival prediction for our study cohort. The marker combinations were used to develop models based on training and testing on the whole cohort. 4 markers: PTEN, SMAD4, CCND1, SPP1. Logit: Logistic Regression. **D)** Kaplan-Meier curve for survival as a function of time generated by a Cox model trained on the whole cohort using the 4 markers. The lowest 2/3 of risk scores was used as threshold for population separation. P value (P) is annotated.

We first tested the platform using a four-protein signature reported in a recent study published by Ding *et al.*[8]. Using a TMA comprised of 405 cases derived from the Physician’s Health Study (PHS), the authors had demonstrated that a multivariate model based on semi-quantitative, pathologist-evaluated protein levels of PTEN, SMAD4, CCND1 and SPP1 could predict lethal outcome. We asked whether we could predict lethal outcome by evaluating protein levels in an independent prostatectomy cohort using our automated system instead. We acquired monoclonal antibodies against all 4 markers and validated them by specificity analyses as described (see Figure 3 and Methods). Out of the 418 qualified cases in our TMA, 340 were found useful for analysis, attrition primarily being due to cores displaced during sectioning (see Table 1 for cohort description). Quantitative tumor epithelium biomarker levels were extracted from each sample and values were subjected to univariate analyses. PTEN, SMAD4 and CCND1 were all found to be individually lethal outcome-predictive with hazard ratios (HRs) of 2.74, 2.48 and 1.99, respectively, while SPP1 did not have significant predictive performance (Figure 4A).

**Table 1:** Composition of patient prostatectomy cohort used in current study

	**# Samples**	**% Samples**	**Num DOD**	**% DOD**
**All**	340	100	35	100
**Gleason 2-6**	124	36.47	2	5.71
**Gleason 7**	127	37.35	4	11.43
**Gleason 8-10**	89	26.18	29	82.86
**Age at diagnosis - mean (SD)**	61.9 (6.7)			
**Median followup years**	11.92			

DOD: dead of disease. SD: standard deviation.

Next, multivariate Cox and logistic regression analyses were conducted. The performance of the four-marker model was determined as an area under the curve (AUC) and a concordance index (CI) (Figure 4B and Additional file 1: Table S3, respectively). For logistic regression analyses, cases were defined as patients that died from prostate cancer. The AUC was approximately 0.75 for the four markers in training mode, and 0.69 to 0.70 in test mode by logistic regression and Cox analyses, respectively (Figure 4B). A Kaplan-Meier curve comparing the top one-third to bottom two-thirds of risk scores based on the four markers was generated by a Cox model trained on the whole cohort. This curve shows a clear survival difference between risk groups (p < 10^-5^, see Figure 4C). Our mean AUC of 0.75 [95% confidence interval (0.67, 0.83)] is comparable with performance as described by Ding *et al.,* with an AUC of 0.83 [95% confidence interval (0.76, 0.91)] [8], as indicated by the large overlap in confidence intervals.

### Incorporation of protein activity states as part of a novel multivariate signature

**Figure 5:**
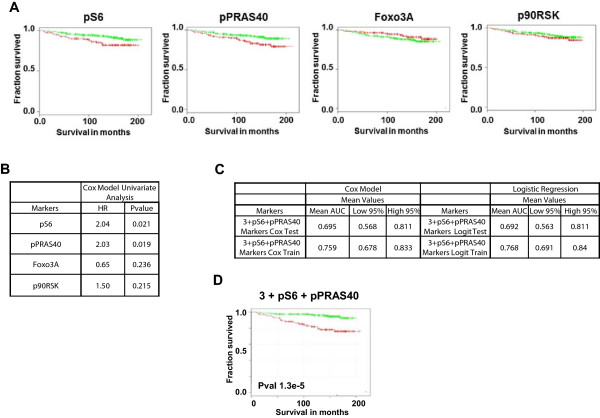
**Phospho markers are predictive of prostate cancer outcome. A)** Kaplan-Meier curves for survival as a function of single biomarker protein expression in the study cohort (pS6, pPRAS40, FOXO3, p90RSK). **B)** Univariate survival analysis. The population with the top 1/3 of risk score values was separated from the population with the bottom 2/3 of risk scores. P values (P) and HR are annotated. **C)** Multivariate Cox regression and logistic regression analyses of survival prediction for our study cohort. The marker combination was used to develop models based on training and testing on the whole cohort. 3 + pS6 + pPRAS40: SMAD4, CCND1, SPP1, pS6, pPRAS40. Logit: Logistic Regression. **D)** Kaplan-Meier curve for survival as a function of risk scores generated by a Cox model trained on the whole cohort using the 3 + pS6 + pPRAS40 markers. The lowest 2/3 of risk scores was used as threshold for population separation. P value (P) is annotated.

Since protein activity states reflect functional events in the tumors that are associated with aggressive behavior, we tested whether our approach could quantitatively measure not just protein levels but protein activity states as reflected by post-translational modifications or altered sub-cellular localization. Phosphorylation is a particularly well-studied example of post-translational modification; the stoichiometry of protein phosphorylation at a particular site is an indirect measure of the activity state of the parent signaling pathway [20,21]. Specifically, we examined whether the activity state of one or more signaling molecules in the core PTEN-regulated signaling pathways PI3K/AKT and MAPK could substitute for PTEN in the four-marker model. PTEN protein, in contrast to the PI3K/AKT pathway, is only altered in a subset of prostate cancers [11,22], so our goal was to identify replacement phosphomarkers that could be more broadly informative about PI3K/AKT pathway activity states [22,23]. To this end, we obtained a number of phospho-specific monoclonal antibodies (mAbs) directed against key phosphoproteins and tested them for technical suitability (Additional file 1: Table S4). Testing included specificity analysis through western blot (WB) and immunohistochemistry (IHC) before and after treatment with the PI3K inhibitor LY294002, signal intensity in human prostate cancer tissue, and, importantly, epitope stability [19,23] based on signal preservation across prostate cancer FFPE samples (Figure 3). We included phospho-markers because PI3K/AKT pathway activity is often independent of PTEN protein status [12,13]. Based on these criteria, the following phospho-specific antibodies were selected and tested for univariate and multivariate lethal outcome predictive performance: p90RSK-T359/S363, pPRAS40-T246, and pS6-S235/236 (Cell Signaling Technology, Danvers, MA [23];). We also selected an anti-FOXO3 antibody for testing since it is excluded from the nucleus when the PI3K pathway is activated [24]. When subjected to univariate analysis, pPRAS40 and pS6 had significant univariate performance with HRs of 2.03 and 2.04, respectively, comparing signal values of the top one-third to bottom two-thirds in a Kaplan-Meier analysis (Figure 5A). The other candidate markers did not reach significance level for univariate performance (Figure 5A).

We next examined the performance of the four original markers without PTEN. The AUC (train) dropped from 0.75 to 0.72-0.73, and addition of either pS6 (in essence substituting pS6 for PTEN) or substitution with pPRAS40 did not result in a significant increase of the AUC and CI, despite their univariate performance (data not shown). However, substitution of PTEN with both pS6 and pPRAS40 increased AUC (train) values to between ~0.76 and ~0.77 (Figure 5B). The corresponding Kaplan-Meier curve for the three markers together with pS6 + pPRAS40 showed significant separation of the top 1/3 from the bottom 2/3 of the cohort (p = 1.3 × 10^-5^; Figure 5C). These results demonstrate that we can successfully replace PTEN, a known lethal outcome-predictive tumor suppressor, with two pathway activity markers, pS6 and pPRAS40, for the development of a new lethal outcome-predictive signature.

## Discussion

The goal of this work was to establish an automated imaging platform that accurately and reproducibly integrates morphological and protein-level information. We assessed platform performance through direct comparison with a previous study by using the same 4 markers reported to predict lethal outcome. While paired data comparing the methods are not available, a simple meta-analysis of the two studies estimates a non-significant difference in mean AUC of 0.08 [95% confidence interval (-0.03, 0.19)]. Differences in performance may be due to methodological differences between the two studies. First, we only used monoclonal antibodies validated for specificity through siRNA oligo-mediated knock down in Western blotting and immunohistochemistry (Figure 3), while two of the antibodies used in the PHS study were polyclonal and thus not ideal for continued prospective application. Moreover, the quantitative measurements in this study were fully automated, while theirs relied on pathologist interpretation, and hence overall would be expected to be slightly less reproducible. Finally, our cohort included a higher proportion of Gleason ≤6 cases for which lethal outcome would be more difficult to predict than for higher grade cases and lethal outcome prediction was further limited by a median follow-up of 11.92 years which is not long enough to capture all deaths. Given these methodological distinctions and the assessment of difference in AUCs, we conclude that our results are comparable, demonstrating an important proof-of-concept for this fully automated platform and prognostication independent of human interpretation.

An important application of this platform is the ability to incorporate protein activation states as biomarkers. The tumor suppressor PTEN, a highly outcome-predictive marker, is altered in only 15-20% of early stage prostate cancers, yet is often functionally inactivated through a variety of other mechanisms that would be reflected in altered PI3K/AKT pathway activity [12]. We here show that phospho-specific mAbs measuring activity states of signaling molecules in the core PI3K and MAPK pathways can substitute for PTEN, and identify pPRAS40 and pS6 as novel, lethal outcome-predictive markers for prostate cancer. The phosphorylation of these markers is directly correlated with the pathway activity state of the PI3K pathway, and both of them are required for both PI3K and MTOR complex 1 (mTORC 1) signaling [23,25]. AKT1S1 (PRAS40) contains a consensus phosphorylation motif and is a direct substrate for AKT, a mediator of PI3K signaling [26], while RPS6 is phosphorylated at Ser235/236 by p70S6 kinase. Interestingly, PRAS40 is required for mTORC 1 signaling to p70S6 kinase, which, in turn, enables p70S6 kinase to phosphorylate RPS6 at Ser235/236. We incorporate these 2 markers into a novel lethal outcome predictive five-marker signature for radical prostatectomy: SMAD4, CCND1, SPP1, pPRAS40 and pS6 and report its performance. To our knowledge, this is the first study that identifies pPRAS40 and pS6 as prognostic markers for prostate cancer.

Over the last few years various quantitative protein-based *in situ* technologies have been developed with varying degrees of success. The pioneering automated quantitative analysis (AQUA) platform for protein measurements is one example [27]. While useful for tissues where single markers can define region of interest, it does not incorporate spectral unmixing and feature extraction capabilities rendering it less suitable for multiplexing and hence problematic for heterogeneous tumors like prostate cancer. Another example is the Aureon platform, which was specifically developed for prostate cancer prognosis [28]. While in some ways similar to the platform we report here, the Aureon platform was developed prior to recent significant advances in automated imaging and biological discoveries in prostate cancer [8]. In this platform, morphological analyses were done on hematoxylin and eosin (H&E)-stained slides, and biomarkers (AMACR and AR) were measured from tissue regions defined by AMACR, a heterogeneously expressed marker present in only ~70-90% of prostate cancer patients, and hence not informative in all cases [16]. Furthermore, full automation was not possible with the first generation imaging software used at the time of development (Definiens Enterprise Image Intelligence Suite [28,29];). Robust tissue segmentation algorithm and quantitative biomarker measurements can now be achieved in tumor epithelium regions by combining Vectra multispectral image decomposition with the programmable Definiens Tissue Developer, The resulting automated approach is highly sensitive, operates without subjective intervention, and can successfully evaluate very small amounts of cancer tissue.

We propose that PI3K/AKT pathway activity state measurements might be more informative in early prostate cancer lesions than PTEN. In ongoing clinical studies on early stage prostate cancer biopsy cohorts we are further testing this notion.

## Conclusions

In summary, we have developed a multiplex immunofluorescence *in situ* imaging platform with automated, objective biomarker measurements able to predict lethal outcome using prostatectomy tissue independent of pathologist interpretation. Importantly, we demonstrate the ability to incorporate quantitative measurements of protein activity states, as reflected by post-translational modifications, into a multivariate protein predictor of lethal outcome, and identify pPRAS40 and pS6 as novel predictive markers for prostate cancer-specific death. We believe that this platform is broadly applicable across disease states. We are currently applying it to the development of a prognostic prostate cancer biopsy test for early stage lesions where tissue amounts are often limited.

## Methods

### Reagents and antibodies

All antibodies and reagents used in this study were procured from commercially available sources as described in Additional file 1: Table S4. Anti-FITC mAb-Alexa568, anti-KRT8-Alexa488, anti-KRT18-Alexa488, anti-KRT5-Alexa555 and anti-TRIM29-Alexa555 were conjugated with Alexa dyes, in-house using appropriate protein conjugation kits, according to manufacturer's instructions (LifeTechnologies, Grand Island, NY).

### Acquisition, processing and quality control of formalin-fixed, paraffin-embedded (FFPE) prostate cancer tissue blocks

We acquired a cohort of FFPE human prostate cancer tissue blocks with clinical annotations and long-term patient outcome information from Folio Biosciences (Powell, OH). Samples had been collected with appropriate institutional review board approval (Phylogeny protocol #001, Quorum Review IRB, 1601 Fifth Avenue, Suite 1000, Seattle WA 98101, file #25552/1) and all patient records were de-identified. We included a number of FFPE human prostate cancer tissue blocks from other commercial sources (BioOptions, Brea, CA; Cureline, So. San Francisco, CA; ILSBio, Chestertown, MD; OriGene, Rockville, MD) to validate individual antibody and combined multiplex staining format staining intensities, to develop quality control procedures, to assess intra-experiment reproducibility studies, and to confirm specificity of staining on prostate tumor tissue.

Between 10 and 12 sections (5 μm cuts) were produced from each FFPE block. The last section was stained with H&E and scanned with an Aperio (Vista, CA) XT system. H&E stained images were deposited into the Spectrum database (Aperio, Vista, CA) for remote reviewing and centralized Gleason annotation in a blinded manner by expert Board-Certified anatomical pathologists using ImageScope software (Aperio, Vista, CA). Annotated circles corresponding to 1 mm cores were placed over four areas of highest and two areas of lowest Gleason patterns on each prostatectomy sample using current criteria (Figure 2A) [30].

### Tissue quality control procedure

A 5 μm section from each FFPE block was stained with anti-pSTAT3 rabbit mAb, anti-STAT3 mouse mAb and region of interest markers, as described below. Slides were examined under a fluorescence microscope. Based on staining intensities and autofluorescence, tissues were qualitatively graded into four categories as shown in Additional file 1: Table S1 and Figure 2A. FFPE blocks belonging to the top two quality categories were included for the study.

### Cell line controls

Selected cell lines to be used as positive and negative controls were grown under standard conditions and treated with drugs and inhibitors before harvesting as indicated (Additional file 1: Table S5). For further details, see Additional file 2: Materials and methods.

### Generation of tumor microarray (TMA) blocks

TMA blocks were prepared using a modified agarose block procedure [31]. Three pairs of TMA blocks (MPTMAF1A and 1B, 2A and 2B, 3A and 3B, respectively) with 91, 170, and 157 annotated prostate tumor samples were constructed (see Additional file 1: Table S2 and Additional file 2: Materials and methods).

A smaller test TMA was generated from commercially available FFPE prostate tumor cases with only limited (Gleason score) annotation. This TMA was used to compare PTEN values with phosphomarkers prior to the main cohort study and to confirm reproducibility. Reproducibility was demonstrated by comparing individual marker signals on consecutive sections of the test TMA (Additional file 1: Table S2 and Figure 2B).

### Slide processing and quantitative multiplex immunofluorescence (QMIF) staining protocol

TMA sections were cut at 5 um thickness and placed on Histogrip (LifeTechnologies, Grand Island, NY) coated slides. Slides were baked at 65°C for 30 min, deparaffinized through serial incubations in xylene, and rehydrated through a series of graded alcohols. Antigen retrieval was performed in 0.05% citraconic anhydride solution for 45 min at 95°C using a PT module (Thermo Scientific, Waltham, MA). Autostainers 360 and 720 (Thermo Scientific, Waltham, MA) were used for staining.The staining procedure involved two blocking steps followed by four incubation steps with appropriate washes in between. Blocking consisted of a biotin step followed by Sniper reagent (Biocare Medical, Concord, CA). The first incubation step included anti-biomarker 1 mouse mAb and anti-biomarker 2 rabbit mAb. The second step included anti-rabbit IgG Fab-FITC and anti-mouse IgG Fab-biotin, followed by a third “visualization” step that included anti-FITC mAb-Alexa568, streptavidin-Alexa633 and fluorophor-conjugated region of interest antibodies (anti-KRT8-Alexa488, anti-KRT18-Alexa488, anti-KRT5-Alexa555 and anti-TRIM29-Alexa555). Finally, sections were incubated with DAPI for nuclear staining (for a staining format outline, see Figure 1B). Slides were mounted with ProlongGold (LifeTechnologies, Grand Island, NY) and coverslipped. Slides were kept at -20°C overnight before imaging and for long-term storage. A full set of 6 MPTMAF slides were stained in a single staining session for the various antibody combinations encompassing all biomarkers tested.

### Antibody validation

mAb specificity was tested by WB before and after knock down. To test the specificity of mAbs against PTEN, SMAD4 and CCND1, we employed inducible shRNA knockdown of the protein markers of interest. Briefly, DU145 cells with inducible shRNA were generated by transducing naïve DU145 cells with a virus carrying pTRIPZ (Thermo Scientific, Waltham, MA). Cells were stably selected using 2 μg/ml puromycin for a week. Subsequently, cells were induced with either 0.1 μg/ml or 2 μg/ml of doxycycline for 72 hours. Cells were trypsinized and processed either for RNA extraction or cell lysate generation. The best shRNA for each protein marker was confirmed first by RT-PCR and then by western blot. Antibodies were considered specific when the expected molecular band size decreased upon shRNA induction on western blot.To test mAb against SPP1, we used cell lines with high or low SPP expression. Lysates from these cell lines (as shown in Figure 3) were also used for Western blotting. The antibody to SPP1 reveals a background band in the BxPC3 cell line that is detectable by western blotting and migrates with a lower apparent molecular weight than SPP1. However, IHC with diamino benzidine (DAB)-based permanent staining against SPP1 reveals almost no detectable background in the BxPC3 cells, confirming the specificity of antibody-based recognition of SPP1. This suggests that the mAb against SPP1 might cross-react with a denatured protein sequence detectable by Western blotting that is not detectable in its native conformation by IHC. The clean background shows that the antibody is highly specific for SPP1 when used for IHC.

To test anti-phospho antibodies against members of the AKT signaling pathway, DU145 cells were serum starved overnight, treated with the PI3K inhibitor LY294002 at 10 μM for 2 hours, and lysed. Lysates from cells treated with inhibitor were used as negative controls for Western blots; lysates from cells grown in standard conditions were used as positive controls.

20 μg of cell lysates were run on a 4-15% Criterion TGX precast gel (Bio-Rad, Hercules, CA). Afterwards, the gel was transferred onto nitrocellulose membrane using iBlot (LifeTechnologies, Grand Island, NY). The primary antibody dilution was used according to product data sheet recommendation. The membrane was developed using SuperSignal West Femto Maximum Sensitivity Substrate (Thermo Scientific, Waltham, MA). Images were captured using the FluroChem Q system (Protein Simple, Santa Clara, CA). Images were processed using AlphaView (Protein Simple, Santa Clara, CA) and ImageJ [32].

For testing by IHC before and after target knock down FFPE cell pellets from cell lines treated as described above were assembled together in a TMA block. 5 μm sections were cut and dried at 60°C for an hour before deparaffinization in three changes of xylene and rehydration in a series of descending ethanol washes. The slides were heated in 0.05% citraconic anhydride (Sigma, Saint Louis, MO) at 95°C for 40 min for antigen retrieval. Slides were stained using the Lab Vision™ UltraVision™ LP Detection System: HRP Polymer/DAB Plus Chromogen Kit (Thermo Scientific, Waltham, MA) as per manufacturer’s instructions. Slides were scanned with an Aperio Scanscope AT Turbo system (Aperio, Vista, CA). Images were analyzed with Aperio ImageScope software (Aperio, Vista, CA).

### Image acquisition

Two Vectra Intelligent Slide Analysis Systems (Perkin-Elmer, Waltham, MA) were used for automated image acquisition. DAPI, FITC, TRITC and Cy5 long pass filter cubes were optimized to allow maximum spectral resolution and minimize cross-interference between fluorophores. Vectra 2.0 and Nuance 2.0 software packages (Perkin Elmer, Waltham, MA) were used for automated image acquisition and development of the spectral library, respectively.

TMA acquisition protocols were run in an automated mode according to manufacturer instructions (Perkin-Elmer, Waltham, MA). Two 20X fields per core were imaged using a multispectral acquisition protocol that included consecutive exposures with DAPI, FITC, TRITC and Cy5 filters. To ensure reproducibility of biomarker quantification, light source intensity was calibrated with the X-Cite Optical Power Measurement System (Lumen Dynamics, Mississauga, ON, Canada) prior to image acquisition for each TMA slide. Identical exposure times were used for all slides containing the same antibody combination. To minimize intra-experiment variability, TMA slides stained with the same antibody combinations were imaged on the same Vectra microscope.A spectral profile was generated for each fluorescent dye as well as for FFPE prostate tissue autofluorescence. Interestingly, two types of autofluorescence were observed in FFPE prostate tissue. A typical autofluorescence signal was common in both benign and tumor tissue, whereas atypical “bright” autofluorescence was specific for bright granules present mostly in epithelial cells of benign tissue. A spectral library containing a combination of these two spectral profiles was used to separate or “unmix” individual dye signals from autofluorescent background (Figure 1A and C).

### Image analysis

We developed an automated image analysis algorithm using Definiens Developer XD (Definiens AG, Munich, Germany) for tumor identification and biomarker quantification. For each 1.0 mm TMA core, two 20X image fields were acquired. Vectra multispectral image files were first converted into multilayer TIFF format using inForm (PerkinElmer, Waltham, MA) and a customized spectral library, then converted to single layer TIFF files using BioFormats (OME [33]). Single layer TIFF files were imported into the Definiens workspace using a customized import algorithm so that for each TMA core, both of the image field TIFF files were loaded and analyzed as “maps” within a single “scene”.Autoadaptive thresholding was used to define fluorescent intensity cut-offs for tissue segmentation in each individual tissue sample. Tissue samples were segmented using DAPI along with fluorescent epithelial and basal cell markers to allow classification as epithelial cells, basal cells and stroma, and were further compartmentalized into cytoplasm and nuclei. Benign prostate glands contain basal cells and luminal cells, whereas prostate cancer glands lack basal cells and have smaller luminal profiles. Therefore, individual gland regions were classified as malignant or benign based on the relational features between basal cells and adjacent epithelial structures combined with object-related features, such as gland size (see Figure 1D). Fields with artifactual staining, insufficient epithelial tissue or out-of-focus images were removed prior to quantification.

Epithelial marker and DAPI intensities were quantitated in benign and malignant epithelial regions as quality control measurements. Biomarker intensity levels were measured in the cytoplasm, nucleus or whole cancer cell based on predetermined subcellular localization criteria. Mean biomarker pixel intensity in the cancer compartments was averaged across maps with acceptable quality parameters to yield a single value for each tissue sample and cell line control core.

### Patient cohort composition

Table 1 describes the composition of the prostatectomy cohort used in the current study.

### Marker value determination

**Figure 6:**
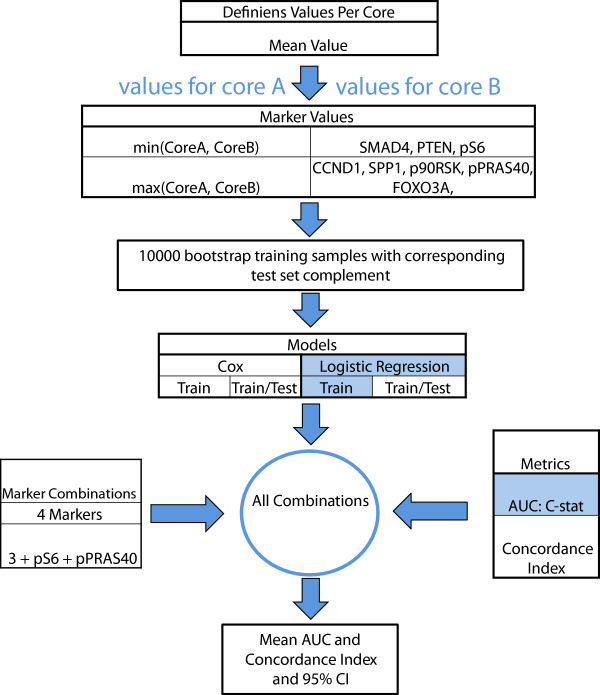
**Outline of statistical analysis flow.** For each patient, two tissue cores from the highest Gleason area were placed into TMA blocks. Mean values of biomarker expression in the tumor epithelium region of each TMA core were used for analysis, resulting in two biomarker values per patient. For PTEN, SMAD4, and pS6, the lowest value from the two cores was used for analysis. For CCND1, SPP1, p90RSK, pPRAS40, and FOXO3, the highest value from the two cores was used. Using these values, 10,000 bootstrap training samples were generated and both multivariate Cox and Logistic Regression models were trained on each training sample. Testing was performed on the complement set. Given the cohort included censored data, we used both CI and AUC to estimate the model performance. The marker combinations that were tested in the models were as follows: 4 markers (PTEN, CCND1, SMAD4, SPP1), 3 markers (CCND1, SMAD4, SPP1), and 3 markers with each of the following combinations of phospho markers: pS6, pPRAS40, and [pS6 + pPRAS40].

As each sample was represented by two cores, we generated an aggregate score for each marker based on correlation direction. For markers correlated positively with lethality we used the core with the highest value; for negatively-correlated markers we used the core with the lowest value. For example, for the tumor suppressor SMAD4, which was present on all stained sections, we used the lowest core value for the two cores (Figure 6).

### Univariate analyses

Univariate cox models were trained for each biomarker. For each marker, the hazard ratio and log rank p-value were calculated to compare the populations consisting of the top one-third and bottom two-thirds of the risk scores for positively correlated markers, and populations consisting of the bottom one-third and top two-thirds of risk scores for negatively correlated markers (Figures 4A and 5A).

### Multivariate analyses

We used multivariate analyses to determine the ability of the marker set to predict lethal outcome. We leveraged two modeling approaches and two metrics. Specifically, 10,000 bootstrap training samples were generated, and both multivariate Cox models and logistic regression models were trained on each training sample. Testing was performed on the complement set. CI and AUC were used to estimate model performance. Kaplan-Meyer curves were generated to compare the population with the bottom two-thirds of risk scores to the population with the top one-third of risk scores. Receiver operating characteristic curves were generated for the whole cohort based on the risk scores from the logistic regression model. Figure 6 presents an outline of the multivariate analysis approaches.

## Abbreviations

AFL: Tissue autofluorescence signals; AQUA: Automated quantitative analysis; AUC: Area under the curve; BAFL: Bright autofluorescence; CI: Concordance index; Cy5: Indodicarbocyanine; DAB: Diamino benzidine; DAPI: Diamidino-2-phenylindole; DOD: Dead of disease; FFPE: Formalin-fixed, paraffin-embedded; FITC: Fluorescein isothiocyanate; H&E: Hematoxylin and eosin; HR: Hazard ratio; IHC: Immunohistochemistry; Logit: Logistic regression; mAb: Monoclonal antibody; MAPK: Mitogen-activated protein kinase; mTORC 1: MTOR complex 1; PHS: Physician’s Health Study; pPRAS40: AKT1S1 phosphorylated on threonine 246; pS6: RPS6 phosphorylated on serines 235/236; pSTAT3: STAT3 phosphorylated on threonine 705; QMIF: Quantitative multiplex immunofluorescence; SD: Standard deviation; TMA: Tumor microarray blocks; TRITC: Tetramethylrhodamine isothiocyanate; WB: Western blotting.

## Competing interests

P.B.-J, M.S., S.C., T.N., Y.H., C.S., S.H., H.C., and E.G. are paid employees and stockholders of Metamark. D.R. is co-founder, stockholder, and consultant for Metamark. D.M.B. is paid consultant for Metamark.

## Authors’ contributions

PB-J conceived the strategy for the automated combined multiplex imaging platform with feature extraction from defined tissue regions, and provided overall guidance for experiments and analyses. MS led and designed all experiments with contributions from CS and DR. Experiments were performed by CS and MS with contributions from SS, antibody validation by YH, SH, and HC. TN developed the image analysis script and workflow. EG conducted all statistical analyses, SC contributed to data analysis, and DMB provided Gleason scoring and tissue diagnosis. PB-J wrote the manuscript with contributions from MS, CS, SC, DMB, DR, and TN. All authors read and approved the final manuscript.

## Supplementary Material

Additional file 1: Table S1 Tissue grading. **Table S2.** TMA Maps. **Table S3.** Concordance index. **Table S4.** Antibodies. **Table S5.** Cell line controls. Click here for file

Additional file 2 Materials and methods. Click here for file
