# Supplementary material for: Automated quantitative multiplex immunofluorescence in situ imaging identifies phospho-S6 and phospho-PRAS40 as predictive protein biomarkers for prostate cancer lethality
Source: Proteome Sci. 2014 Jul 12;12:40. doi: 10.1186/1477-5956-12-40 (PMC4114438; doi:10.1186/1477-5956-12-40)
Supplement: Additional file 2 — Materials and methods. [file 1477-5956-12-40-S2.docx]

**Additional file**

**Materials and Methods**

**Cell line controls**

Selected cell lines to be used as positive and negative controls were grown under standard conditions and treated with drugs and inhibitors before harvesting as indicated (Supplemental Table 4). Cells were washed with phosphate-buffered saline (PBS), fixed directly on plates with 10% formalin for 5 min, then scraped and collected into PBS. Next, cells were washed twice with PBS, resuspended in Histogel (Thermo Scientific, Waltham, MA) at 70^0^ C, and spun for 5 minutes (10,000 g) to form a condensed cell-Histogel pellet. Pellets were embedded in paraffin, placed into standard paraffin blocks, and used as donor blocks for tumor microarray construction.

**Generation of tumor microarray (TMA) blocks**

Agarose blocks (0.7%) were embedded into paraffin and used as TMA acceptor blocks. Using a TMA Master (3DHistech, Budapest, Hungary) instrument, two 1 mm diameter cores were drilled into donor blocks from areas corresponding to the highest Gleason pattern according to pathologist annotation. One of these cores was placed in a randomized position in one acceptor block while the position of the other core in a second acceptor block was randomized relative to the first core. This was repeated with 91, 170 and 157 annotated prostate tumor samples (Supplemental Table 3) to form 3 pairs of TMA blocks (MPTMAF1A and 1B, 2A and 2B, 3A and 3B) respectively. The resulting paired blocks were identical in terms of patient sample composition but randomized in terms of sample position. Cell line control cores were added to top, middle and bottom portions of these acceptor blocks. Once loaded, TMA blocks were placed face down on glass slides at 65^0^C for 15 min to enable fusion of TMA cores into host paraffin. Paraffin blocks were then cut into 5 μm serial sections.
